# Improving Compliance and Satisfaction With Quality Coffee Intake to Enhance Bowel Recovery After Colorectal Surgery: A Feasibility Study

**DOI:** 10.1002/hsr2.71724

**Published:** 2026-01-08

**Authors:** Constant Delabays, Fabian Grass, Nicolas Demartines, Dieter Hahnloser, Martin Hübner

**Affiliations:** ^1^ Department of Visceral Surgery, Lausanne University Hospital CHUV University of Lausanne (UNIL) Lausanne Switzerland

**Keywords:** bowel recovery, coffee, colorectal surgery, compliance, enhanced recovery, ileus

## Abstract

**Background and Aim:**

Coffee after digestive surgery is a simple, safe, and inexpensive prevention for post‐operative ileus (POI). Several meta‐analyses have shown that coffee consumption in the post‐operative period may enhance bowel recovery. However, uptake in clinical routine is variable, standardization challenging and compliance unknown. We hypothesized that serving tasty and freshly made quality coffee will enhance compliance and facilitate implementation of coffee following surgery.

**Methods:**

In this prospective monocentric single arm study, we proposed to every patient undergoing elective colorectal surgery 3 daily doses of freshly made and coffee with a capsule system over 3 days. Patient could choose between four different flavors with standardized caffeine level. Primary endpoints were compliance (x/total possible doses) and reasons for non‐compliance. Secondary outcome was satisfaction of patients using a visual analogue scale (VAS: 0–10).

**Results:**

50 patients were included for analysis. Median postoperative coffee consumption was 6 (IQR 5–8) during the first three post‐operative days (POD), and was not modified after exclusion of patients with POI (*n* = 5). Median compliance was 78% (IQR 56–100) and 35 patients (70%) drank more than 2/3 of possible doses. Compliance decreased post‐operatively by POD 2. The main reason for non‐compliance was nausea (*n* = 28). Patients' satisfaction with the coffee was high (mean VAS 8.2 ± 1.6).

**Conclusion:**

This pilot study revealed high compliance and satisfaction with a standardized, personalized and freshly brewed coffee together with an enjoyable moment. Combining therapeutic interventions with enjoyable experiences may represent a promising approach to increased compliance to standardized postoperative recovery measures.

**Abbreviations**
ERAS: enhanced recovery after surgery
IQR: interquartile range
POD: Post‐operative day
POI: Post‐operative ileus
PREM: Patient‐reported experience measures
RCT: randomised controlled trial
VAS: visual analogic scale

## 1 Introduction

Colorectal surgery, even performed by minimally invasive techniques, is frequently followed by postoperative ileus (POI). This condition affects about 20% of patients after elective colorectal surgery and causes a lot of discomfort [1, 2]. In addition, POI results in prolonged hospital stay and significantly increased healthcare costs [3, 4]. Resumption of bowel function is a key component of recovery and can be achieved by a multimodal approach such as enhanced recovery after surgery (ERAS).

Coffee is a very popular beverage and the most consumed pharmacological substance worldwide [5]. Coffee has proven benefits on general well‐being, especially due to its effect on central nervous and cardiovascular systems [6, 7, 8]. Coffee has a well‐established effect on gastrointestinal motility, even though the exact mechanism is unknown. Recent studies demonstrated that drinking coffee in the post‐operative period leads to a faster recovery of bowel function after colorectal surgery, without any associated adverse effect, nor increased morbidity [9, 10, 11, 12, 13]. These results were further validated by several meta‐analyses [14, 15, 16, 17]. Even if the evidence is limited due to methodological shortcomings of these trials (substantial heterogenicity, single center design) resulting in a low to moderate level of evidence, coffee is emerging as a simple, safe and inexpensive measure for POI prevention.

However, compliance was shown to be particularly challenging for the introduction of oral supplements and drinks in ERAS protocols [18, 19]. Furthermore, taste and sensory experience are elements influencing compliance with nutritional products. It is common knowledge that coffee served at the hospital is not the most pleasant experience, and no data about compliance in clinical settings is available. Hospital coffee is either prepared in advance, often in large quantity, with instant coffee, making caffeine level very variable, which implies a lack of standardization. Moreover, during production of soluble coffee, spray or freeze drying inevitably drive off and remove a significant proportion of the volatile compounds that give coffee its characteristic aroma. In contrast capsule systems hermetically seal volatile aromas for over 12 months [20, 21]. This aroma conservation was repeatedly reflected by blind tasting award, consistently assigning higher scores to capsule blends. Thus, we suggest that the key to ensure widespread implementation and routine use of a standardized coffee for POI prevention is to choose a freshly made quality coffee “at the patient's bedside”, according to personal preference (flavour and amount).

The aims of this study were to measure patients' compliance and satisfaction with tasty, freshly made and high‐quality coffee with a capsule system to enhance compliance for POI prevention after colorectal surgery.

## 2 Methods

### 2.1 Study Protocol and Participants

The study was designed as a prospective monocentric single arm feasibility study analysing compliance with freshly made coffee after colorectal surgery. From November 2021 to August 2022, all patients aged 18 years and older and scheduled for elective colorectal surgery or ostomy closure, were included in the study. Written informed consent was obtained until the day before surgery. Patients were excluded if they had impaired cognitive status, communication problems, pre‐existing ileus, or need for post‐operative surveillance in intensive or intermediate care units. We did not propose the protocol to patients with known hypersensitivity or allergy to coffee, nor to pregnant women. Inclusion to the study ended when a total of 50 patients with complete data was reached. The sample size of 50 patients was empirically defined according to previous feasibility studies to assess compliance to evidence‐based interventions [19].

### 2.2 Objectives and Endpoints

The main objective of this study was to evaluate standardized and systematic implementation of serving a tasty and freshly made coffee into clinical practice in the post‐operative period. The primary endpoint was therefore compliance to coffee from a capsule system at the suggested schedule as well as reasons for non‐compliance. Intake of 2/3 per day was considered compliant in line with ERAS recommendations and previous studies about compliance with nutritional products [18, 22]. Compliance with coffee was also compared to compliance with other ERAS items. Secondary endpoints were satisfaction and the experience felt for each participant using a specific and dedicated questionnaire (patient‐related experience measure, PREM). The latter was conducted in two parts: the first part evaluated satisfaction using a VAS (Visual Analogic Scale) from 1 to 10, and the second part consisted of closed‐ended questions (yes or no) about the experience. Other secondary outcomes included bowel recovery (time to first flatus and first bowel movement), length of hospital stay (interval from day of operation until day of discharge), and post‐operative 30‐day complication. However, the effect of improved compliance on bowel recovery was not specifically assessed in the setting of this pilot study. Complications were graded using the validated Clavien classification [23].

### 2.3 Study Intervention

Daily doses of freshly made coffee with capsule system were proposed to participants 3 times a day during the first 3 postoperative days. Coffee intake was not limited, and participants could take supplementary doses should their usual consumption exceed the suggested schedule. The choice for the capsule coffee system was based on four criteria: (I) standardized caffeine level, (II) availability, (III) different and enjoyable flavours, (IV) easy to use. Doses were scheduled on a standard basis (8:00 a.m., 12:00 p.m. and 3:00 p.m. in accordance with meal services) but remained flexible and could be adapted according to the patients' preferences. This protocol and schedule is based on studies demonstrating the effectiveness of coffee intake in POI prevention [9, 12]. In addition, participants were encouraged to use the coffee machine themselves, thus stimulating early mobilization. The participant could choose among a selection of four different types of coffee ensuring a variety of flavours to satisfy different tastes and add a ludic dimension. Moreover, the coffee was offered in two ways: Espresso (40 mL) or Lungo (110 mL), at the patients' discretion. The dose of caffeine was standardized in each capsule, varying between 60 and 80 mg depending on the type and variety of coffee. Milk and sugar were at patient's free disposal to refine the individual gustatory experience. Finally, in order to guarantee an optimal sensory and visual experience, coffee was offered in glass cups.

Before, during and after surgery, patients were followed according to the standard of care institutional ERAS program adopted by our unit [24, 25]. This program improves patients' convalescence by actively involving them, reducing surgical stress and shortening hospitalization time [22, 26, 27, 28, 29].

### 2.4 Ethics

The current study was conducted in accordance with the Declaration of Helsinki. It was approved and audited by the local ethics committee (CER‐VD 2021‐014430). Written informed consent was obtained from all subjects involved in this study until the day before surgery. The study funder had no role in the study design, data collection, analysis, interpretation, manuscript preparation and decision to publish the results.

### 2.5 Statistical Analysis

Continuous variables were presented as median and interquartile range or mean with standard deviation, and categorical variables as absolute number and percentage. We used descriptive statistics, including *t* tests for continuous variables and *χ* ^2^ for discrete variables. Statistical significance was defined as a two‐sided *p* value less than 0.05. Data analysis was performed with the Statistical Software for the Social Sciences SPSS Advanced Statistics 29 (IBM Software Group, 200 W. Madison St., Chicago, IL; 60606 USA) and Microsoft Excel (version 16.16.27). Study data were collected and managed using REDCap electronic data capture tools hosted at CHUV. REDCap (Research Electronic Data Capture) is a secure, web‐based software platform designed to support data capture for research studies, providing I) an intuitive interface for validated data capture; II) audit trails for tracking data manipulation and export procedures; III) automated export procedures for seamless data downloads to common statistical packages; and IV) procedures for data integration and interoperability with external sources [30, 31].

## 3 Results

### 3.1 Patients and Surgical Characteristics

**Figure 1:**
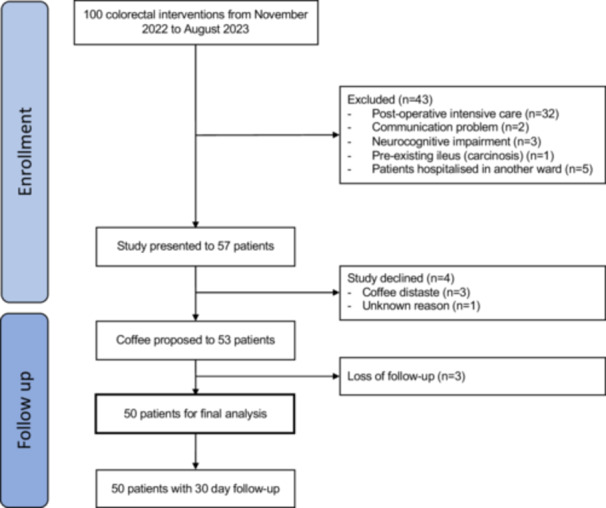
Flow diagram for the study.

From November 2021 to August 2022, a total of 100 patients undergoing elective colorectal interventions were screened (Figure 1). The study was proposed to 57 eligible patients and only four patients refused to participate, mainly because of coffee distaste (*n* = 3). Participation rate was therefore high (93%). Three patients were excluded from final analysis due to missing data. Baseline demographics and surgical characteristics are listed in Table 1. Forty‐seven patients (94%) consumed coffee on a daily basis before the operation, and 60% of the cohort (*n* = 35) drank three coffees or more before the intervention. About a third (*n* = 14, 28%) were smokers, and all of them were coffee consumers. The most frequently performed procedure was left colectomy (*n* = 18, 36%), and the main indication for surgery was colorectal cancer (*n* = 26, 52%). Most interventions were laparoscopic or robotic assisted (*n* = 37, 74%) with a low conversion rate (*n* = 1, 2.7%).

**Table 1:** Demographic and surgical characteristics of all patients (*n* = 50).

**Demographics**	
Age, median (IQR)	61 (44–66)
Male gender (%)	29 (58)
**Habitus**	
Smoker (%)	14 (28)
Smoke habits	
None	36
< 10	2 (4)
10–20	7 (14)
21–30	3 (6)
31–40	2 (4)
Daily regular coffee consumer (%)	47 (94)
Pre‐operative coffee intake (cup(s) per day)	
None (%)	3 (6)
1–2 cups (%)	17 (34)
3–4 cups (%)	26 (52)
≥ 5 cups (%)	4 (8)
Alcohol overconsumer (%)	5 (10)
**Comorbidities**	
Diabetes	5 (10)
Cardiovascular disease (%)	19 (3)
Respiratory disease (%)	4 (8)
BMI (kg/m^2^, mean +/−SD)	26.3 ± 4.8
ASA class ≥ 3 (%)	13 (26)
**Indication for surgery**	
Colonic cancer (%)	14 (28)
Rectal cancer (%)	12 (24)
Diverticular disease (%)	7 (14)
Inflammatory bowel disease (%)	3 (6)
Other (%)	14 (28)
**Procedure**	
Sigmoidectomy and left colectomy (%)	18 (36)
Low anterior rectal resection and abdomino‐perineal amputation (%)	11 (22)
Ostomy closure (ileostomy, colostomy and Hartmann reversal) (%)	11 (22)
Right colectomy (%)	9 (18)
Other (%)	1 (2)
**Operative characeteristics**	
Laparoscopic (%)	22 (44)
Robotic (%)	15 (30)
Open	4 (8)
Direct approach (ostomy)	9 (18)
Operative time (min, mean ± SD)	180 ± 84

*Note:* Continuous variables are presented as median ± interquartile range (IQR) or mean ± standard deviation. Nominal variables are presented as absolute number and percent. Alcohol overconsumption was defined as ≥ 14U/week. ASA, American Society of Anesthesiologists; BMI, Body Mass Index; min, minutes.

### 3.2 Coffee Compliance

When analysing the size of coffee, patients preferred Espresso (40 mL) rather than Lungo (100 mL) (*n* = 208, 69% vs *n* = 95, 31%). Flavours were equivalently chosen, with a slight preference for fruity and medium intensity coffee (Table S1 and Figure S1). Only two patients took additional coffee, and only on the first POD (post‐operative day).

**Figure 2:**
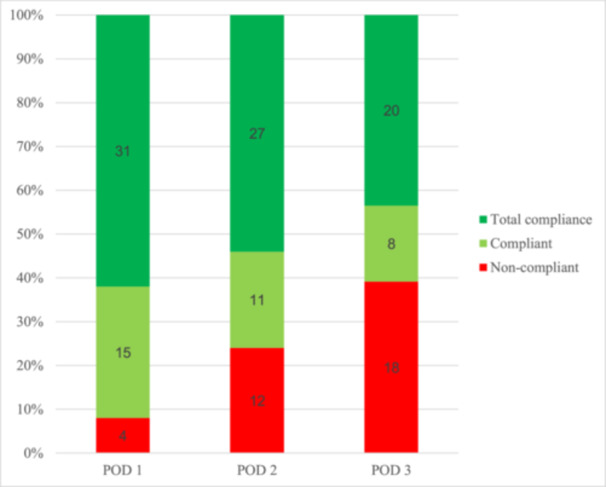
Compliance. The figure displays compliance to the suggested coffee schedule (3×/day). Compliance was calculated as actual intake compared to the maximal possible dose (= total compliance). Patients who consumed two or more doses (> 66%) were considered as compliant (green columns). Absolute number of patients displayed within columns. POD, Post‐operative day.

**Figure 3:**
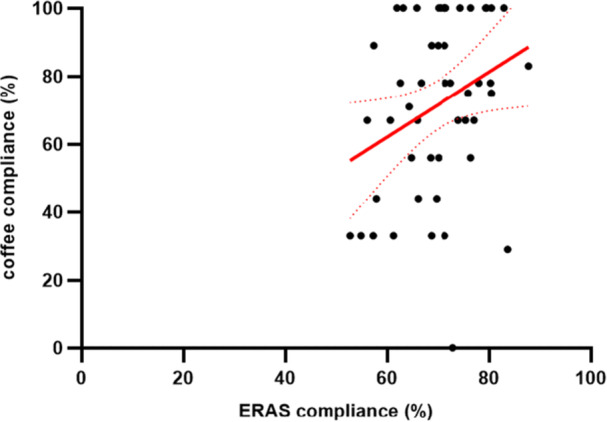
Scatter Plot comparing compliance of ERAS items to coffee compliance. Y‐axis represents coffee compliance and x‐axis overall compliance to ERAS items. Compliance with coffee intake correlated with overall compliance to ERAS items (*ρ* = 0.299).

Twelve patients were discharged before post‐operative day 3, and could not consume all nine coffees. Compliance was therefore calculated based on the maximal possible amount of coffee during hospitalisation for each patient. Median overall coffee consumption between POD 1–3 was 6 [5, 6, 7, 8] and median coffee compliance rate was 78% (IQR 56–100). 35 patients (70%) drank more than 2/3 of possible doses during the study. Nine patients (18%) drank 9/9 portions and 15 patients (30%) drank all possible doses. When analysing coffee adherence to the protocol throughout the study, coffee consumption decreased from POD1‐3 and satisfying compliance (> 2/3) was observed in respectively 92%, 76%, and 61% of patients (Figure 2). Compliance with coffee intake correlated with overall compliance to ERAS items (*ρ* = 0.299, Figure 3). We conducted subgroup analysis to explore demographic determinant of compliance. Although we saw some evidence of improved response rates in men when compared with women (78% ± 23 vs 64% ± 28), differences between groups did not meet conventional levels of statistical significance (*p* = 0.07). Compliance did not vary by smoking status, age ( < 65 vs. ≥ 65 years), ASA class ( < 3 vs. ≥ 3), or coffee intake ( ≤ 2 vs. ≥ 3 cups/day).

Patients drank a total of 303 doses out of 422 possible occasions (71.8%). Reasons for non‐compliance are detailed in Table 2. Nausea was the main reason, accounting for 24% of missed coffees. Lack of compliance due to staff happened 8 times, accounting for 7% of missed doses. Five patients (10%) presented with POI during the first three POD and needed nasogastric tube reinsertion. Those patients could not drink further coffee, and missed a total of 21 coffees. Median coffee consumption was however similar after exclusion of these patients.

**Table 2:** Reasons for non‐compliance for each missed coffee dose (*n*, %).

Nausea	28 (24)
Ileus with gastric tube	21 (18)
Patient not in the mood	13 (11)
Unknown reason	13 (11)
Two coffees are enough	11 (9)
Coffee not proposed by nurse	8 (7)
Disgused	6 (5)
Pain	5 (4)
Too late for coffee	5 (4)
One coffee is enough	5 (4)
Too much nursing	1 (1)
Fatigue	1 (1)
Problem with machine	1 (1)
Other reason	1 (1)
Total	119

*Note:* The table shows reasons for non‐compliance among all patients (*n* = 50) provided by patients in the diary after each missed dose. They are expressed in absolute number and percentage (total of 119 missed doses).

### 3.3 Secondary Outcomes

**Figure 4:**
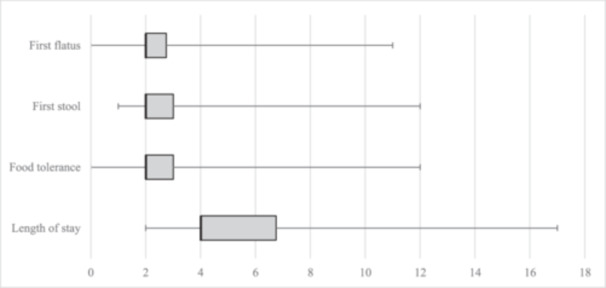
Whisker plot representing secondary outcomes. Whisker plot with main secondary outcomes on y‐axis. X‐axis expresses post‐operative nights. Median time (bold lines), interquartile range (whisker plots), and range (lines) are displayed.

Bowel recovery and hospital stay are depicted in Figure 4. Thirty percent of patients (*n* = 15) presented at least one post‐operative complication, as resumed in Table 3. Major complications were observed in four patients. Nine patients (18%) developed a POI and needed nasogastric tube reinsertion. Median duration of nasogastric drainage was 3 days (IQR 2–4).

**Table 3:** Secondary Outcomes (*n*, %).

Morbidity at 30 days	
Any complication	23 (46)
Patient with at least one complication	15 (30)
Minor complication ( < III) (%)	19
Major complication ( ≥ III) (%)	4
I (%)	2 (4)
II (%)	17 (34)
IIIA (%)	3 (6)
IIIB (%)	1 (2)
IV (%)	0 (0)
Reinsertion of nasogastric tube ( = POI)	9 (18)
Median time of gastric drainage in days (IQR)	3 (2–3)
**Mortality at 30 days (%)**	0 (0)
**Readmission (%)**	1 (2)

*Note:* The table shows secondary outcome of the study population (*n* = 50 patients). Complication were graded using the validated Clavien classification. Major complications were defined as grade III to V. Values are presented as absolute number and percentage. POI: postoperative ileus; Values. IQR, Interquartile range.

### 3.4 Patient‐Reported Experience Measures

**Figure 5:**
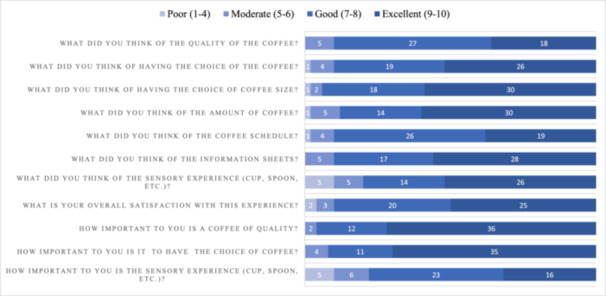
Satisfaction with coffee protocol. Patient satisfaction with coffee protocol. Patients were instructed to quote each item from 1 to 10, with a visual analogic scale. Results were then divided in four categories: (I) excellent, (II) good, (III) moderate and poor (IV).

All included patients completed the satisfaction questionnaire. Results are summarised in Figure 5. Satisfaction was very high concerning the quality, the choice of taste and size of the coffee, as well as the amount of coffee and schedule of doses. Global satisfaction with the experience was very good (mean score of 8.2 ± 1.6). It was very important for participants to have a coffee of good quality (mean score 8.9 ± 1.1) and to have the choice among different flavours (mean score 8.8 ± 1.3. Sensory experience was important for most patients (mean score 7.3 ± 2.2). 42% (*n* = 21) of participants were aware of the benefits of coffee on general health, but almost none (*n* = 1, 2%) knew the positive effects on postoperative bowel recovery. This awareness had a positive impact for 58% (*n* = 29) of our patients in the post‐operative period and motivated coffee consumption. Among patients who were already hospitalized before (*n* = 32), one third indicated to drink more coffee during this present stay. Ninety‐one percent (*n* = 29) noticed a difference in the taste of coffee, which was better during this study. Eighty‐six percent (*n* = 43) of patients would like to benefit again from freshly brewed coffee in case of a further hospitalisation.

## 4 Discussion

In the present study, colorectal surgery patients had good compliance and excellent satisfaction and experience metrics with a personalized coffee protocol offering freshly made coffee of various flavours in enjoyable cups. Furthermore, patients reported a very high level of satisfaction and emphasize benefits of such a protocol compared to regular hospital coffee.

Several Randomized Controlled Trials (RCT) and systematic review with meta‐analyses of RCT demonstrated cconsistently accelerated gastrointestinal recovery after abdominal and colorectal surgery, despite methodological limitations related to heterogeneity and study design [9, 10, 11, 12, 32, 33]. However, these studies were conducted in rigorous and controlled settings, where coffee consumption was closely monitored and recorded by medical staff, which does not represent daily clinical practice. High compliance rate is necessary in order to achieve similar results and to benefit the patients. This study is to our knowledge the first to specifically explore and refine compliance and satisfaction to coffee in the post‐operative setting. Although our study was not designed to directly assess the impact of improved coffee consumption on the incidence of POI, our findings contribute to a better understanding of how increased awareness to standardized and evidence‐based measures may impact on postoperative recovery through improved compliance and satisfaction.

Patient's awareness, presence of symptoms, socio‐economic status and ethnicity, complexity and frequency of the treatment are some of many aspects shaping compliance [34]. Satisfaction, taste and experience of a substance can strongly affect its consumption, particularly for nutritional products [18]. A tasteful and enjoyable coffee was therefore crucial to analyse compliance to evidence‐based recommendations in this present study. Offering a high‐quality product may be of particular importance after colorectal surgery, where patients frequently suffer from nausea, abdominal discomfort, and dysgeusia, which can strongly affect oral intake. Hence, focusing on the sensory experiment of a cup of coffee may be an effective measure to achieve better compliance.

Patient awareness is an important factor influencing compliance. Understanding the benefits of coffee intake during the post‐operative period is crucial to increase its consumption and to seek the clinical benefit. Nearly all participants (98%) were not aware of the positive impact of coffee on bowel function recovery at the time of study inclusion. Oral and written information provided increased awareness and motivated 58% of patients to drink more coffee and therefore strongly contribute to achieve high compliance. Information remains one of the key elements for sustained compliance of enhanced recovery programs, especially in the post‐operative period when active patient involvement is needed and where the thrilling concept of patient's empowerment makes perfect sense. Furthermore, both machine and capsule were at patient disposal in dedicated locations in the surgical ward. Patients were instructed to walk and serve themselves whenever possible, in order to stimulate early mobilisation, integral part of the ERAS protocol.

Coffee is a complex chemical mixture containing hundreds of various compounds [6]. The way coffee affects gastrointestinal function is yet still not fully understood. The RCT by Müller et al compared the effect of coffee versus warm water after colonic resection and showed a decreased time to first bowel movement [9]. Those results were confirmed by Hasler et al, who found the same results when compared to decaffeinated tea after laparoscopic colectomy [12]. A study from Piric et al showed similar results, with moreover a reduced length of stay and complication rate [11]. However, in the latter, patients had open colectomy, and post‐operative care did not follow an enhance recovery protocol, which no longer reflects best clinical practice. Benefits of coffee in that setting might be overestimated. An interesting study by Dulskas et al compared coffee with decaffeinated coffee and water after colonic resection [10]. Remarkably, they found a significantly stronger effect of decaffeinated coffee, suggesting that caffeine might not be the main active compound helping with gastrointestinal functional recovery [10]. This finding is corroborate by a recent RCT, which showed no benefits of caffeine capsules compared to placebo after laparoscopic colectomy [35]. Those results suggest that other coffee components rather than caffeine might promote bowel recovery, and that decaffeination may form new chemically active agents. Other active substances in coffee have been suggested to participate in colonic peristaltic activity such as chlorogenic acids and melanoidins [36, 37].

The use of a capsule‐based coffee system introduced a level of standardization that was not achievable with soluble coffee preparations, which is essential for future research. While it would be challenging to isolate and demonstrate any direct effect of the capsule system itself on gastrointestinal motility compared to soluble coffee, we believe that the primary advantage of this approach lies in its potential to improve patient compliance, in line with ERAS recommendations. Before large scale implementation, further studies are needed to explore cost effectiveness and workflow integration.

Some limitations deserve consideration. First, the sample size is limited and the population derives from one single center, over a discrete period of time. Hence, the study should be considered a preliminary, exploratory investigation rather than a dedicated, adequately powered trial. Statistical analysis is limited, but 50 participants are adequate for a feasibility study. Exclusion criteria and need for intensive/intermediate care in particular induce a risk for selection bias. Some potential confounders such as pre‐existing nausea and vomiting, the extent of surgery or bowel resection, and sociocultural demographics were either not available or could not be analyzed as subgroups due to the small sample size of this pilot study. However, participation rate was high, and the study population is likely to reflect clinical practice of a “real‐world”, busy and heterogeneous colorectal surgery practice. Finally, coffee consumption, quality assessment and taste are subjective metrics and cannot be uncritically extrapolated. Furthermore, different preparation preferences (milk, ice, adjuncts) may have an impact on caffeine content and individual desirability.

Participants did not report adverse effects of coffee such as tachyarrhythmia or sleep disturbance. This is supported by a recent meta‐analysis including 13 RCTs totalling 1246 patients after abdominal surgery, which found no adverse effects related to postoperative coffee consumption [15]. Finally, the recently published CaCo trial analysed the effect of caffeine capsules compared to placebo. Secondary outcomes included quality of sleep and found that patients in the study arm woke up significantly more often but experienced less headache than placebo. Caffeine capsules however were distributed with meals, also for dinner. A better schedule at the patient's discretion might help to prevent such adverse effects. The stimulating effect of caffeine on early mobilisation could also be further investigated.

## 5 Conclusion

Compliance and satisfaction with a standardized coffee intake by use of a simple to use capsule system was high. Serving a personalized coffee (flavour, size and schedule), together with a pleasant sensory experience such as can be experience in a restaurant, can increase compliance up to 78%. Expresso (40 mL) with moderate intensity and fruity flavours was the preferred blend in our population. Combining therapeutic interventions with enjoyable experiences may represent a promising approach to increased compliance to post‐operative measures and could be extended to other ERAS items.

## Author Contributions

**Constant Delabays:** conceptualization, methodology, software, formal analysis, investigation, resources, data curation, writing – original draft, visualization, writing – review and editing. **Fabian Grass:** conceptualization, methodology, software, formal analysis, resources, writing – review and editing, visualization, supervision, project administration. **Nicolas Demartines:** resources, writing – review and editing, supervision, funding acquisition. **Dieter Hahnloser:** resources, writing – review and editing. **Martin Hübner:** conceptualization, methodology, supervision, funding acquisition, project administration, resources, writing – review and editing.

## Ethics Statement

The study was conducted in accordance with the Declaration of Helsinki, and approved by the local Ethics Committee (CER‐VD 2021‐014430) for studies involving humans.

## Consent

Informed consent was obtained from all subjects involved in the study.

## Conflicts of Interest

The authors declare no conflicts of interest.

## Transparency Statement

Constant Delabays affirms that this manuscript is an honest, accurate, and transparent account of the study being reported; that no important aspects of the study have been omitted; and that any discrepancies from the study as planned have been explained.

## Supporting information

Supplementary Materials.pdf.

## Data Availability

The data that support the findings of this study are available from the corresponding author upon reasonable request.
