# Supplementary material for: Improving Compliance and Satisfaction With Quality Coffee Intake to Enhance Bowel Recovery After Colorectal Surgery: A Feasibility Study
Source: Health Sci Rep. 2026 Jan 8;9(1):e71724. doi: 10.1002/hsr2.71724 (PMC12783215; doi:10.1002/hsr2.71724)

Supplementary Materials

Table S1: Characteristics of the different available flavours

	**Description**	**Intensity**	**Caffein (mg/cup)**	
			**Espresso**	**Lungo**
**Indonesia**	Woody	8	72	81
**Ethiopia**	Fruity, Winey	4	63	74
**Nicaragua**	Sweet, Honey	5	65	77
**Columbia**	Fruity, Winey	6	69	76

Caffein level is expressed as mg per cup, either espresso (40ml) or lungo (110ml)

Figure S1: Pie chart representing size (A) and flavours (B) of selected coffees

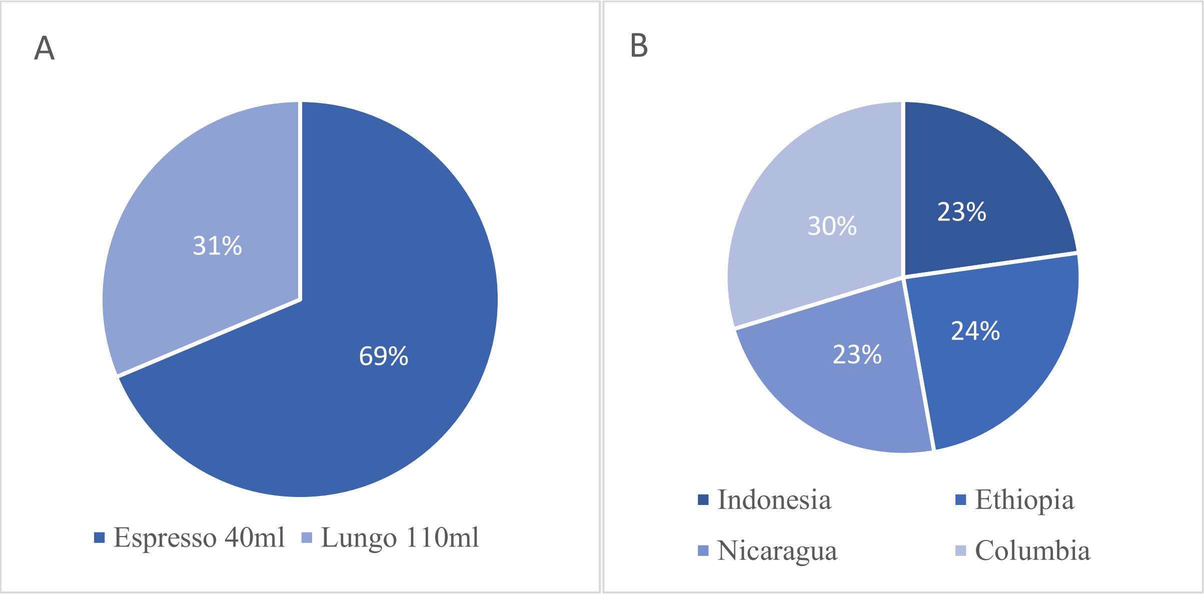

Supplement: Supplementary file 1 — Supplementary Materials.pdf. [file HSR2-9-e71724-s001.pdf]
